# MRI strain analysis as a novel modality for the assessment of myocardial function following stem cell therapy-results from Amorcyte trial

**DOI:** 10.1186/1532-429X-13-S1-P86

**Published:** 2011-02-02

**Authors:** Sabha Bhatti, Abdul Hakeem, Michael Taylor, Eugene Chung, Arshed A Quyyumi, John Oshinski, Andrew L Pecora, Dean Kereiakes, Kan Hor, Wojciech Mazur

**Affiliations:** 1 University of Cincinnati Hospital, Cincinnati, OH, USA; 2 Cincinnati Childrens Hospital, Cincinnati, OH, USA; 3 The Christ Hospital, Cincinnati, OH, USA; 4 Emory University, Atlanta, GA, USA; 5 Amorcyte Inc, Hackensack, NJ, USA

## Objective

To evaluate MRI strain analysis for the assessment of myocardial function following stem cell therapy.

## Background

MRI strain analysis is a novel way to assess myocardial function and may detect subtle improvements in myocardial function earlier than commonly used methods of myocardial function assessment. Stem cell therapy offers a promising approach to the regeneration of damaged vascular and cardiac tissue after myocardial infarction. Myocardial strain imaging has not been evaluated as an end point in stem cell studies. Our objective was to demonstrate the role and feasibility of MRI strain analysis for the early detection of myocardial functional improvement following stem cell therapy.

## Methods

The Amorcyte trial randomized 31 patients to an autologous stem cell harvest group (cells were harvested from the patient’s own bone marrow) or control group five days after an ST elevation myocardial infarction (87% Anterior). This is the the first randomized study to prospectively define a dose of a purified and potent autologous stem cell therapy that resulted in a significant improvement in perfusion, a trend towards improved EF (+4% versus +1%). Using the TomTec research arena MRI software ©, circumferential strain was measured in short axis views at base, mid and apex for each patient at baseline, 3 and 6 months.

## Results

Thirteen patients in the treatment and 11 controls were available for strain analysis. There was no significant difference in the two groups with respect to baseline characteristics including age 52+8 vs. 53+10.5 (p= 0.7), gender, race, mean ejection fraction, NYHA class, hypertension and revascularization. Mean apical circumferential strain at baseline was -17.2 (95% CI-22.2, -12.1) and increased to -20.6 (95% CI -26.7,-14.5) at 6 months (p=0.03).Mid anterior circumferential strain showed a strong trend to improvement between baseline -17(95%CI -23.6,-10.4) and 3 months -20.49(95% CI -25,-15.7) p=0.05. There was no significant change in the base at 3 month and 6 months compared to baseline for other segments. Comparatively, no significant change was noted in segmental circumferential strain in the control group.

## Conclusions

Despite a small patient population in whom traditional methods of cardiac function only showed a trend toward improvement with stem cell therapy, MRI was able to demonstrate a significant increment in circumferential strain in the apex and mid anterior segments. Larger studies evaluating MRI derived strain analysis as an endpoint following stem cell therapy including correlation with clinical endpoints are warranted.

